# (1*R*,3*S*,4*R*,4a*S*,7*R*,7a*S*,10*R*,12a*R*)-3-Azido-4,7,10-trimethyl-1,10-epidioxy­per­hydropyrano[4,3-*j*][1,2]benzodiox­epine

**DOI:** 10.1107/S1600536810024566

**Published:** 2010-06-26

**Authors:** Lijun Xie, Xin Zhai, Jian Zuo, Yanfang Zhao, Ping Gong

**Affiliations:** a Key Laboratory of Original New Drug Design and Discovery of the Ministry of Education, Shenyang Pharmaceutical University, Shenyang, Liaoning 110016, People’s Republic of China; b Key Laboratory of Marine Chemistry Theory and Technology, Ministry of Education, College of Chemistry and Chemical Engineering, Ocean University of China, Qingdao, Shandong 266100, People’s Republic of China

## Abstract

In the title compound, C_15_H_23_N_3_O_4_, the six-membered pyran, cyclo­hexane and trioxane rings adopt chair, chair and boat conformations, respectively, while the seven-membered rings adopt distorted boat and very distorted chair conformations. In the crystal, adjacent mol­ecules are connected by weak C—H⋯N and C—H⋯O inter­actions.

## Related literature

For general background to artemisinin, a sesquiterpene endoperoxide widely used to treat drug-resistant malaria, see: Liu *et al.* (1979 ▶). For the anti­cancer properties of the title compound, see: Efferth *et al.* (1996 ▶); Chadwick *et al.* (2009 ▶); Galal *et al.* (2009 ▶). For structural analyses of highly related compounds, see: Gul *et al.* (2009 ▶); Jasinski*et al.* (2008 ▶).
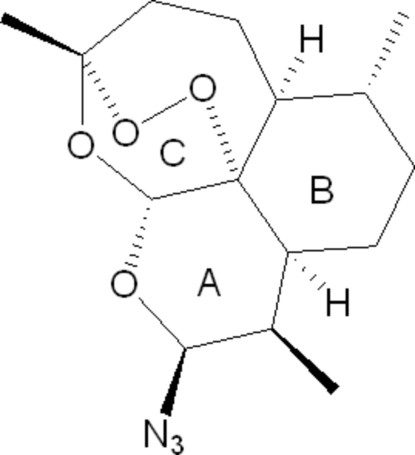


## Experimental

### 

#### Crystal data

C_15_H_23_N_3_O_4_
*M* *_r_* = 309.36
Orthorhombic, 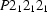

*a* = 7.9938 (9) Å
*b* = 11.207 (1) Å
*c* = 17.984 (2) Å
*V* = 1611.1 (3) Å^3^
*Z* = 4
Mo *K*α radiation
μ = 0.09 mm^−1^
*T* = 298 K
0.50 × 0.40 × 0.38 mm

#### Data collection

Bruker SMART CCD area-detector diffractometer
Absorption correction: multi-scan (*SADABS*; Sheldrick, 1996 ▶) *T*_min_ = 0.955, *T*_max_ = 0.965
7585 measured reflections
1657 independent reflections
1130 reflections with *I* > 2σ(*I*)
*R*_int_ = 0.043

#### Refinement

*R*[*F* ^2^ > 2σ(*F* ^2^)] = 0.040
*wR*(*F* ^2^) = 0.103
*S* = 1.09
1657 reflections
202 parameters
H-atom parameters constrained
Δρ_max_ = 0.12 e Å^−3^
Δρ_min_ = −0.16 e Å^−3^

### 

Data collection: *SMART* (Bruker, 2003 ▶); cell refinement: *SAINT* (Bruker, 2003 ▶); data reduction: *SAINT*; program(s) used to solve structure: *SHELXS97* (Sheldrick, 2008 ▶); program(s) used to refine structure: *SHELXL97* (Sheldrick, 2008 ▶); molecular graphics: *SHELXTL* (Sheldrick, 2008 ▶); software used to prepare material for publication: *SHELXTL*.

## Supplementary Material

Crystal structure: contains datablocks I, global. DOI: 10.1107/S1600536810024566/im2212sup1.cif

Structure factors: contains datablocks I. DOI: 10.1107/S1600536810024566/im2212Isup2.hkl

Additional supplementary materials:  crystallographic information; 3D view; checkCIF report

## Figures and Tables

**Table 1:** Hydrogen-bond geometry (Å, °)

*D*—H⋯*A*	*D*—H	H⋯*A*	*D*⋯*A*	*D*—H⋯*A*
C7—H7*B*⋯N3^i^	0.97	2.68	3.628 (6)	167
C10—H10⋯O3^ii^	0.98	2.67	3.535 (5)	148
C12—H12*A*⋯O3^ii^	0.97	2.65	3.508 (5)	147

Symmetry codes: (i) 
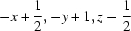
; (ii) 
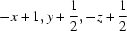
.

**Fig. 1.**
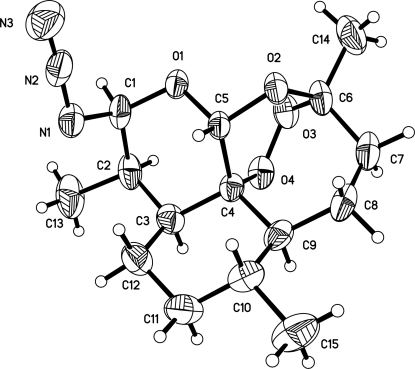
Molecular structure of the title compound, showing 30% probability displacement ellipsoids and the atom-numbering scheme.

## supplementary crystallographic information

### Comment

Artemisinin, a sesquiterpene endoperoxide isolated from Artemisia annua *L*, is being widely used to treat drug-resistant malaria (Liu *et al.*, 1979). In addition, Artemisinin and its derivatives also showed potent and broad anticancer properties in different human cancer cell lines and animal models (Efferth *et al.*, 1996). These compounds contain an endoperoxide bridge (R—O—O—*R*) which is required for their biological activities. Recently, there are many reports about significant anticancer activities of artemisinin derivatives, which were expected to be more stable toward the metabolism process (Chadwick *et al.*, 2009; Galal *et al.*, 2009). Herein, we present the synthesis and structure of an artemisinin derivatives, (1*R*,3S,4*R*,4aS,7*R*,7aS,10*R*,12aR)-3-Azido- 4,7,10-trimethyl-1,10-epoxy-decahydro- 12*H*-pyrano[4,3-*j*]-1,2-benzodioxepin.

The crystal structure of the title compound is given in Fig. 1. The bond lengths and angles in the title compound are found to have normal values with respect to highly related compounds (Gul *et al.*, 2009; Jasinski *et al.*, 2008). The six membered rings A, B and C adopt chair, chair and boat conformations, respectively. In the crystal, adjacent molecules are connected by non-classical C—H···N and C—H···O hydrogen bonding, with the distance of 3.628 (6), 3.508 (5) and 3.535 (5) Å (Table 1), respectively.

### Experimental

Trimethylchlorosilane (300 mmol, 38.1 ml) was added gradually to a solution of dihydroartemisinin (200 mmol, 56.8 g, diastereomeric mixture with *R* and *S* configuration at C(3)) and sodium azide (300 mmol, 19.5 g) in CH_2_Cl_2 _(300 ml). Then sodium iodide (20 mmol, 3.0 g) was added to the reaction mixture at low temperature. The reaction mixture was stirred at room temperature for 28 h. The mixture was quenched with a saturated NaHCO_3_ solution (100 ml) and diluted with CH_2_Cl_2_. Two phases were separated and the organic phase was washed with brine, dried over MgSO_4_, filtered, and concentrated under reduced pressure. The crude mixture was purified by column chromatography (silica, 1%-5% EtOAc/hexanes) to furnish the product (94 mmol, 29.0 g) and it's diastereomer with *R* configuration at C(3). Colorless single crystals of the title compound was obtained in CH_2_Cl_2_ solution after 10 days by slow evaporation at room temperature.

### Refinement

In the absence of significant anomalous dispersion effects, Friedel pairs were averaged. All H-atoms were positioned geometrically and refined using a riding model, with C—H = 0.96 Å (CH_3_), 0.97 Å (CH_2_), 0.98 Å (CH), and *U*_iso_(H) =1.2*U*_eq_(C).

### Crystal data

**Table:**

C_15_H_23_N_3_O_4_	*F*(000) = 664
*M**_r_* = 309.36	*D*_x_ = 1.275 Mg m^−^^3^
Orthorhombic, *P*2_1_2_1_2_1_	Mo *K*α radiation, λ = 0.71073 Å
Hall symbol: P 2ac 2ab	Cell parameters from 2309 reflections
*a* = 7.9938 (9) Å	θ = 2.3–21.4°
*b* = 11.207 (1) Å	µ = 0.09 mm^−^^1^
*c* = 17.984 (2) Å	*T* = 298 K
*V* = 1611.1 (3) Å^3^	Block, colorless
*Z* = 4	0.50 × 0.40 × 0.38 mm

### Data collection

**Table:**

Bruker SMART CCD area-detector diffractometer	1657 independent reflections
Radiation source: fine-focus sealed tube	1130 reflections with *I* > 2σ(*I*)
graphite	*R*_int_ = 0.043
phi and ω scans	θ_max_ = 25.0°, θ_min_ = 2.1°
Absorption correction: multi-scan (*SADABS*; Sheldrick, 1996)	*h* = −9→7
*T*_min_ = 0.955, *T*_max_ = 0.965	*k* = −13→13
7585 measured reflections	*l* = −16→21

### Refinement

**Table:**

Refinement on *F*^2^	Primary atom site location: structure-invariant direct methods
Least-squares matrix: full	Secondary atom site location: difference Fourier map
*R*[*F*^2^ > 2σ(*F*^2^)] = 0.040	Hydrogen site location: inferred from neighbouring sites
*wR*(*F*^2^) = 0.103	H-atom parameters constrained
*S* = 1.09	*w* = 1/[σ^2^(*F*_o_^2^) + (0.0312*P*)^2^ + 0.4488*P*] where *P* = (*F*_o_^2^ + 2*F*_c_^2^)/3
1657 reflections	(Δ/σ)_max_ < 0.001
202 parameters	Δρ_max_ = 0.12 e Å^−^^3^
0 restraints	Δρ_min_ = −0.16 e Å^−^^3^

### Special details

**Table:**

Experimental. We took dihydroartemisinin (mixture of 3R and 3S isomers of hydroxyl group) as the starting material in our experiment. During the course of synthesis, we got a mixture of two diastereomers with 3S and 3R and all other stereogenic centers are known and still in the configuration as they were in the starting compound. The mixture was separated by silica gel column chromatography and the title compound with 3S was crystallized under our conditions, while the other one (3R) was obtained as amorphous powder.
Geometry. All e.s.d.'s (except the e.s.d. in the dihedral angle between two l.s. planes) are estimated using the full covariance matrix. The cell e.s.d.'s are taken into account individually in the estimation of e.s.d.'s in distances, angles and torsion angles; correlations between e.s.d.'s in cell parameters are only used when they are defined by crystal symmetry. An approximate (isotropic) treatment of cell e.s.d.'s is used for estimating e.s.d.'s involving l.s. planes.
Refinement. Refinement of *F*^2^ against ALL reflections. The weighted *R*-factor *wR* and goodness of fit *S* are based on *F*^2^, conventional *R*-factors *R* are based on *F*, with *F* set to zero for negative *F*^2^. The threshold expression of *F*^2^ > σ(*F*^2^) is used only for calculating *R*-factors(gt) *etc*. and is not relevant to the choice of reflections for refinement. *R*-factors based on *F*^2^ are statistically about twice as large as those based on *F*, and *R*- factors based on ALL data will be even larger.

### Fractional atomic coordinates and isotropic or equivalent isotropic displacement parameters (Å^2^)

**Table:**

	*x*	*y*	*z*	*U*_iso_*/*U*_eq_	
N1	0.4489 (5)	0.4264 (3)	0.41235 (17)	0.0737 (10)	
N2	0.3162 (6)	0.4810 (3)	0.41854 (19)	0.0756 (10)	
N3	0.2016 (6)	0.5395 (4)	0.4253 (3)	0.1117 (15)	
O1	0.3528 (3)	0.3297 (2)	0.30143 (11)	0.0583 (6)	
O2	0.3573 (3)	0.3910 (2)	0.18223 (12)	0.0601 (7)	
O3	0.4236 (3)	0.1895 (2)	0.17929 (14)	0.0726 (8)	
O4	0.5935 (3)	0.2044 (2)	0.20749 (13)	0.0669 (7)	
C1	0.4297 (5)	0.3114 (3)	0.37062 (18)	0.0656 (10)	
H1	0.3554	0.2601	0.3999	0.079*	
C2	0.5957 (5)	0.2491 (4)	0.3652 (2)	0.0738 (12)	
H2	0.5712	0.1695	0.3454	0.089*	
C3	0.7131 (5)	0.3075 (4)	0.3087 (2)	0.0670 (11)	
H3	0.8021	0.2495	0.2989	0.080*	
C4	0.6214 (4)	0.3259 (3)	0.23485 (17)	0.0528 (9)	
C5	0.4547 (4)	0.3878 (3)	0.24650 (17)	0.0495 (9)	
H5	0.4759	0.4699	0.2627	0.059*	
C6	0.3823 (5)	0.2889 (4)	0.13467 (19)	0.0683 (11)	
C7	0.5171 (5)	0.3170 (4)	0.0778 (2)	0.0789 (12)	
H7A	0.5780	0.2444	0.0667	0.095*	
H7B	0.4638	0.3435	0.0322	0.095*	
C8	0.6406 (6)	0.4117 (4)	0.1028 (2)	0.0765 (12)	
H8A	0.5809	0.4867	0.1075	0.092*	
H8B	0.7234	0.4218	0.0639	0.092*	
C9	0.7325 (5)	0.3881 (3)	0.1758 (2)	0.0653 (10)	
H9	0.8227	0.3319	0.1640	0.078*	
C10	0.8171 (5)	0.5007 (4)	0.2052 (2)	0.0798 (12)	
H10	0.7293	0.5586	0.2172	0.096*	
C11	0.9123 (5)	0.4740 (5)	0.2759 (3)	0.0973 (16)	
H11A	0.9621	0.5470	0.2945	0.117*	
H11B	1.0020	0.4184	0.2650	0.117*	
C12	0.7998 (5)	0.4211 (4)	0.3354 (2)	0.0840 (13)	
H12A	0.7159	0.4795	0.3493	0.101*	
H12B	0.8661	0.4032	0.3791	0.101*	
C13	0.6724 (7)	0.2282 (5)	0.4424 (2)	0.1150 (19)	
H13A	0.6936	0.3037	0.4659	0.173*	
H13B	0.7756	0.1851	0.4374	0.173*	
H13C	0.5960	0.1828	0.4724	0.173*	
C14	0.2153 (6)	0.2572 (5)	0.1011 (3)	0.1060 (17)	
H14A	0.2293	0.1929	0.0665	0.159*	
H14B	0.1704	0.3255	0.0758	0.159*	
H14C	0.1398	0.2332	0.1398	0.159*	
C15	0.9352 (6)	0.5587 (5)	0.1476 (3)	0.124 (2)	
H15A	0.9829	0.6300	0.1683	0.186*	
H15B	0.8730	0.5785	0.1036	0.186*	
H15C	1.0229	0.5038	0.1351	0.186*	

### Atomic displacement parameters (Å^2^)

**Table:**

	*U*^11^	*U*^22^	*U*^33^	*U*^12^	*U*^13^	*U*^23^
N1	0.085 (2)	0.083 (2)	0.0530 (19)	−0.002 (2)	0.003 (2)	−0.0156 (19)
N2	0.094 (3)	0.079 (3)	0.055 (2)	−0.016 (2)	0.017 (2)	−0.015 (2)
N3	0.105 (3)	0.112 (3)	0.117 (4)	0.005 (3)	0.021 (3)	−0.037 (3)
O1	0.0652 (14)	0.0705 (16)	0.0393 (12)	−0.0044 (13)	0.0041 (11)	−0.0011 (12)
O2	0.0722 (15)	0.0672 (15)	0.0410 (13)	0.0191 (14)	−0.0084 (13)	−0.0062 (13)
O3	0.101 (2)	0.0608 (16)	0.0560 (14)	−0.0006 (16)	−0.0084 (15)	−0.0067 (15)
O4	0.0912 (19)	0.0523 (15)	0.0572 (14)	0.0182 (14)	0.0006 (14)	−0.0024 (13)
C1	0.095 (3)	0.065 (2)	0.0366 (17)	−0.013 (2)	0.0044 (19)	−0.0005 (19)
C2	0.107 (3)	0.070 (3)	0.044 (2)	0.014 (3)	−0.011 (2)	0.005 (2)
C3	0.068 (2)	0.071 (3)	0.062 (2)	0.019 (2)	−0.010 (2)	0.002 (2)
C4	0.061 (2)	0.051 (2)	0.0465 (18)	0.0126 (19)	0.0015 (17)	−0.0009 (17)
C5	0.058 (2)	0.054 (2)	0.0362 (16)	0.0069 (19)	−0.0015 (18)	−0.0046 (17)
C6	0.094 (3)	0.068 (3)	0.0431 (19)	0.013 (2)	−0.011 (2)	−0.008 (2)
C7	0.111 (3)	0.083 (3)	0.043 (2)	0.025 (3)	0.003 (2)	−0.002 (2)
C8	0.099 (3)	0.082 (3)	0.048 (2)	0.013 (3)	0.022 (2)	0.010 (2)
C9	0.065 (2)	0.068 (2)	0.063 (2)	0.017 (2)	0.016 (2)	−0.001 (2)
C10	0.067 (3)	0.083 (3)	0.090 (3)	−0.003 (2)	0.016 (2)	0.000 (3)
C11	0.063 (3)	0.118 (4)	0.111 (4)	−0.008 (3)	−0.001 (3)	−0.011 (3)
C12	0.076 (3)	0.103 (3)	0.073 (3)	0.007 (3)	−0.018 (2)	−0.006 (3)
C13	0.160 (5)	0.127 (4)	0.057 (3)	0.039 (4)	−0.024 (3)	0.018 (3)
C14	0.120 (4)	0.124 (4)	0.074 (3)	0.003 (4)	−0.035 (3)	−0.030 (3)
C15	0.108 (4)	0.121 (4)	0.143 (5)	−0.028 (4)	0.040 (4)	0.010 (4)

### Geometric parameters (Å, °)

**Table:**

N1—N2	1.229 (5)	C7—H7A	0.9700
N1—C1	1.499 (5)	C7—H7B	0.9700
N2—N3	1.133 (5)	C8—C9	1.528 (5)
O1—C1	1.403 (4)	C8—H8A	0.9700
O1—C5	1.437 (4)	C8—H8B	0.9700
O2—C5	1.394 (4)	C9—C10	1.526 (5)
O2—C6	1.442 (4)	C9—H9	0.9800
O3—C6	1.412 (4)	C10—C11	1.511 (6)
O3—O4	1.459 (3)	C10—C15	1.545 (6)
O4—C4	1.464 (4)	C10—H10	0.9800
C1—C2	1.503 (6)	C11—C12	1.518 (6)
C1—H1	0.9800	C11—H11A	0.9700
C2—C3	1.530 (5)	C11—H11B	0.9700
C2—C13	1.536 (5)	C12—H12A	0.9700
C2—H2	0.9800	C12—H12B	0.9700
C3—C12	1.527 (5)	C13—H13A	0.9600
C3—C4	1.531 (4)	C13—H13B	0.9600
C3—H3	0.9800	C13—H13C	0.9600
C4—C5	1.517 (5)	C14—H14A	0.9600
C4—C9	1.550 (5)	C14—H14B	0.9600
C5—H5	0.9800	C14—H14C	0.9600
C6—C14	1.507 (5)	C15—H15A	0.9600
C6—C7	1.518 (5)	C15—H15B	0.9600
C7—C8	1.518 (6)	C15—H15C	0.9600
			
N2—N1—C1	112.6 (3)	C7—C8—C9	116.5 (3)
N3—N2—N1	174.3 (4)	C7—C8—H8A	108.2
C1—O1—C5	115.3 (3)	C9—C8—H8A	108.2
C5—O2—C6	113.2 (3)	C7—C8—H8B	108.2
C6—O3—O4	108.9 (3)	C9—C8—H8B	108.2
O3—O4—C4	111.4 (2)	H8A—C8—H8B	107.3
O1—C1—N1	111.3 (3)	C10—C9—C8	111.6 (3)
O1—C1—C2	113.4 (3)	C10—C9—C4	112.8 (3)
N1—C1—C2	110.0 (3)	C8—C9—C4	113.0 (3)
O1—C1—H1	107.3	C10—C9—H9	106.3
N1—C1—H1	107.3	C8—C9—H9	106.3
C2—C1—H1	107.3	C4—C9—H9	106.3
C1—C2—C3	112.7 (3)	C11—C10—C9	110.6 (4)
C1—C2—C13	111.4 (3)	C11—C10—C15	109.9 (4)
C3—C2—C13	114.9 (4)	C9—C10—C15	112.7 (4)
C1—C2—H2	105.7	C11—C10—H10	107.9
C3—C2—H2	105.7	C9—C10—H10	107.9
C13—C2—H2	105.7	C15—C10—H10	107.9
C12—C3—C2	115.3 (3)	C10—C11—C12	111.8 (3)
C12—C3—C4	112.2 (3)	C10—C11—H11A	109.3
C2—C3—C4	109.9 (3)	C12—C11—H11A	109.3
C12—C3—H3	106.3	C10—C11—H11B	109.3
C2—C3—H3	106.3	C12—C11—H11B	109.3
C4—C3—H3	106.3	H11A—C11—H11B	107.9
O4—C4—C5	109.7 (3)	C11—C12—C3	111.9 (4)
O4—C4—C3	103.9 (3)	C11—C12—H12A	109.2
C5—C4—C3	111.2 (3)	C3—C12—H12A	109.2
O4—C4—C9	106.0 (3)	C11—C12—H12B	109.2
C5—C4—C9	113.1 (3)	C3—C12—H12B	109.2
C3—C4—C9	112.4 (3)	H12A—C12—H12B	107.9
O2—C5—O1	105.4 (3)	C2—C13—H13A	109.5
O2—C5—C4	112.8 (3)	C2—C13—H13B	109.5
O1—C5—C4	112.7 (3)	H13A—C13—H13B	109.5
O2—C5—H5	108.6	C2—C13—H13C	109.5
O1—C5—H5	108.6	H13A—C13—H13C	109.5
C4—C5—H5	108.6	H13B—C13—H13C	109.5
O3—C6—O2	108.7 (3)	C6—C14—H14A	109.5
O3—C6—C14	104.4 (4)	C6—C14—H14B	109.5
O2—C6—C14	107.5 (3)	H14A—C14—H14B	109.5
O3—C6—C7	112.4 (3)	C6—C14—H14C	109.5
O2—C6—C7	109.5 (3)	H14A—C14—H14C	109.5
C14—C6—C7	114.1 (3)	H14B—C14—H14C	109.5
C8—C7—C6	114.0 (3)	C10—C15—H15A	109.5
C8—C7—H7A	108.7	C10—C15—H15B	109.5
C6—C7—H7A	108.7	H15A—C15—H15B	109.5
C8—C7—H7B	108.7	C10—C15—H15C	109.5
C6—C7—H7B	108.7	H15A—C15—H15C	109.5
H7A—C7—H7B	107.6	H15B—C15—H15C	109.5
			
C1—N1—N2—N3	−159 (4)	O4—C4—C5—O1	62.1 (3)
C6—O3—O4—C4	44.7 (3)	C3—C4—C5—O1	−52.3 (4)
C5—O1—C1—N1	72.1 (4)	C9—C4—C5—O1	−179.9 (3)
C5—O1—C1—C2	−52.6 (4)	O4—O3—C6—O2	−72.3 (3)
N2—N1—C1—O1	53.7 (4)	O4—O3—C6—C14	173.2 (3)
N2—N1—C1—C2	−179.8 (3)	O4—O3—C6—C7	49.0 (4)
O1—C1—C2—C3	50.7 (4)	C5—O2—C6—O3	30.7 (4)
N1—C1—C2—C3	−74.6 (4)	C5—O2—C6—C14	143.2 (3)
O1—C1—C2—C13	−178.5 (4)	C5—O2—C6—C7	−92.4 (3)
N1—C1—C2—C13	56.2 (5)	O3—C6—C7—C8	−95.2 (4)
C1—C2—C3—C12	78.1 (4)	O2—C6—C7—C8	25.7 (4)
C13—C2—C3—C12	−50.9 (5)	C14—C6—C7—C8	146.2 (4)
C1—C2—C3—C4	−49.8 (4)	C6—C7—C8—C9	56.2 (5)
C13—C2—C3—C4	−178.8 (3)	C7—C8—C9—C10	−165.3 (3)
O3—O4—C4—C5	16.4 (3)	C7—C8—C9—C4	−36.8 (5)
O3—O4—C4—C3	135.4 (3)	O4—C4—C9—C10	−162.7 (3)
O3—O4—C4—C9	−106.0 (3)	C5—C4—C9—C10	77.1 (4)
C12—C3—C4—O4	162.9 (3)	C3—C4—C9—C10	−49.8 (4)
C2—C3—C4—O4	−67.5 (4)	O4—C4—C9—C8	69.6 (4)
C12—C3—C4—C5	−79.1 (4)	C5—C4—C9—C8	−50.7 (4)
C2—C3—C4—C5	50.5 (4)	C3—C4—C9—C8	−177.6 (3)
C12—C3—C4—C9	48.8 (4)	C8—C9—C10—C11	−177.9 (3)
C2—C3—C4—C9	178.4 (3)	C4—C9—C10—C11	53.5 (4)
C6—O2—C5—O1	−91.5 (3)	C8—C9—C10—C15	−54.6 (5)
C6—O2—C5—C4	31.9 (4)	C4—C9—C10—C15	176.9 (3)
C1—O1—C5—O2	177.1 (3)	C9—C10—C11—C12	−57.2 (5)
C1—O1—C5—C4	53.7 (4)	C15—C10—C11—C12	177.8 (4)
O4—C4—C5—O2	−57.1 (4)	C10—C11—C12—C3	57.2 (5)
C3—C4—C5—O2	−171.5 (3)	C2—C3—C12—C11	−179.4 (3)
C9—C4—C5—O2	61.0 (4)	C4—C3—C12—C11	−52.6 (4)

### Hydrogen-bond geometry (Å, °)

**Table:**

*D*—H···*A*	*D*—H	H···*A*	*D*···*A*	*D*—H···*A*
C7—H7B···N3^i^	0.97	2.68	3.628 (6)	167
C10—H10···O3^ii^	0.98	2.67	3.535 (5)	148
C12—H12A···O3^ii^	0.97	2.65	3.508 (5)	147

Symmetry codes: (i) −*x*+1/2, −*y*+1, *z*−1/2; (ii) −*x*+1, *y*+1/2, −*z*+1/2.
